# Sleep, circadian rhythm, and physical activity patterns in depressive and anxiety disorders: A 2‐week ambulatory assessment study

**DOI:** 10.1002/da.22949

**Published:** 2019-07-26

**Authors:** Sonia Difrancesco, Femke Lamers, Harriëtte Riese, Kathleen R. Merikangas, Aartjan T. F. Beekman, Albert M. van Hemert, Robert A. Schoevers, Brenda W. J. H. Penninx

**Affiliations:** ^1^ Amsterdam UMC, Vrije Universiteit, Psychiatry Amsterdam Public Health Research Institute Amsterdam The Netherlands; ^2^ University of Groningen, University Medical Center Groningen, Department of Psychiatry Interdisciplinary Center for Psychopathology and Emotion Regulation Groningen The Netherlands; ^3^ Genetic Epidemiology Branch, Intramural Research Program National Institute of Mental Health Bethesda Maryland; ^4^ Department of Psychiatry Leiden University Medical Center Leiden The Netherlands

**Keywords:** actigraphy, anxiety disorders, circadian rhythm, comorbidity, depressive disorder, dysthymic disorder, exercise, sleep

## Abstract

**Background:**

Actigraphy may provide a more valid assessment of sleep, circadian rhythm (CR), and physical activity (PA) than self‐reported questionnaires, but has not been used widely to study the association with depression/anxiety and their clinical characteristics.

**Methods:**

Fourteen‐day actigraphy data of 359 participants with current (*n* = 93), remitted (*n* = 176), or no (*n* = 90) composite international diagnostic interview depression/anxiety diagnoses were obtained from the Netherlands Study of Depression and Anxiety. Objective estimates included sleep duration (SD), sleep efficiency, relative amplitude (RA) between day‐time and night‐time activity, mid sleep on free days (MSF), gross motor activity (GMA), and moderate‐to‐vigorous PA (MVPA). Self‐reported measures included insomnia rating scale, SD, MSF, metabolic equivalent total, and MVPA.

**Results:**

Compared to controls, individuals with current depression/anxiety had a significantly different objective, but not self‐reported, PA and CR: lower GMA (23.83 vs. 27.4 milli‐gravity/day, *p* = .022), lower MVPA (35.32 vs. 47.64 min/day, *p* = .023), lower RA (0.82 vs. 0.83, *p* = .033). In contrast, self‐reported, but not objective, sleep differed between people with current depression/anxiety compared to those without current disorders; people with current depression/anxiety reported both shorter and longer SD and more insomnia. More depressive/anxiety symptoms and number of depressive/anxiety diagnoses were associated with larger disturbances of the actigraphy measures.

**Conclusion:**

Actigraphy provides ecologically valid information on sleep, CR, and PA that enhances data from self‐reported questionnaires. As those with more severe or comorbid forms showed the lowest PA and most CR disruptions, the potential for adjunctive behavioral and chronotherapy interventions should be explored, as well as the potential of actigraphy to monitor treatment response to such interventions.

## 1 INTRODUCTION

Depression and anxiety are highly prevalent psychiatric disorders, with largely overlapping pathophysiology (Zorn et al., 2017), high genetic correlation (Wray, 2018), both causing high disability (Vos et al., 2016) and sharing high degree of comorbidity (Lamers et al., 2011; Rodney et al., 1997). Sleep and circadian rhythm (CR) disturbances and altered physical activity (PA) have long been recognized as core features of depression and anxiety.

Studies have shown that insomnia and hypersomnia are more frequent among those with a diagnosis of depression (Nutt, Wilson, & Paterson, 2008; van Mill, Hoogendijk, Vogelzangs, van Dyck, & Penninx, 2010), and comorbid depression and anxiety (van Mill et al., 2010), which is not surprising as experiencing insomnia or hypersomnia nearly every day is one of the DSM‐5 diagnostic criteria of depressive disorders. But more insomnia and hypersomnia have also been reported in anxiety disorders (Staner, 2003; van Mill et al., 2010). Evening chronotype has been linked to depressive (Antypa, Vogelzangs, Meesters, Schoevers, & Penninx, 2016; Norbury, 2019) and anxiety disorders (Kivelä, Papadopoulos, & Antypa, 2018), depressive symptoms as reported in the meta‐analysis by Au and Reece (2017) and to comorbid depressive and anxiety disorders (Antypa et al., 2016). Reduced PA has been reported in patients with depression as shown in the meta‐analysis by Schuch et al. (2017), in patients with anxiety (Hiles, Lamers, Milaneschi, & Penninx, 2017; Ströhle, 2009) and in patients with comorbid depressive and anxiety disorders (Hiles et al., 2017). These findings are mainly based on retrospective self‐reported questionnaires, that summarize static estimates, which are potentially biased by patient's cognitive impairments and negative perception (Sallis & Saelens, 2000).

Wrist‐worn actigraphy devices can objectively assess (disturbances in) sleep, CR, and PA. The ecological measurement with actigraphy in patients’ natural environments continuously over time may translate much more readily to potentially effective intervention (Shiffman, Stone, & Hufford, 2008). For instance, actigraphy may be used for clinical assessment to monitor treatment outcomes. Although actigraphy has become widely available in epidemiological research (Doherty et al., 2017), only few large‐cohort studies have studied the relationship of sleep, CR, and PA with depression and anxiety in a psychiatric sample using actigraphy. In addition, there is limited research studying the association of objective estimates with clinical characteristics, such as psychiatric comorbidity and chronicity of psychiatric disorders. Thus, a better understanding on the association of sleep, CR, and PA with psychopathology that may have important clinical implications, in particular for the clinical assessment, monitoring, and treatment of depression and anxiety.

In this study, we investigate whether (a) objective and/or self‐reported estimates of sleep, CR, and PA differ in persons without and with remitted or current depressive and/or anxiety disorders; and (b) objective estimates of PA, sleep, and CR are associated with clinical characteristics (i.e., severity of symptoms, number of psychiatric disorders, duration of psychiatric disorders, age of onset, and antidepressant use). As part of the objective, (a) we also explore the correlations between objective and self‐reported estimates of sleep, CR, and PA. This may give us an insight whether the more objective actigraphy collection is truly providing novel information.

## 2 METHODS

### 2.1 Sample

Participants from the Netherlands Study of Depression and Anxiety (NESDA) were selected to participate in the Ecological Momentary Assessment (EMA) & Actigraphy sub‐study (NESDA‐EMAA). NESDA is one of the sites that is a member of the Motor Activity Research Consortium for Health (m‐MARCH; Scott et al., 2017), a collaborative network on the application of objective assessment of motor activity, sleep, and mood in population and clinical samples. Details about NESDA have been provided extensively before (Penninx et al., 2008). NESDA was designed to investigate the course of depressive and anxiety disorders over a period of several years and the factors that influence the development and prognosis of such disorders. Participants were initially included at the baseline assessment in 2004 to 2007 (n = 2981), and seen for the fifth time at the 9‐year follow‐up assessment wave (2014–2017) for a regular follow‐up interview (n = 1776), including a psychiatric diagnostic interview. Siblings of a subsample of NESDA participants were (also) included by asking NESDA participants at the regular 9‐year follow‐up interview for their consent to approach their siblings. Eligibility criteria were that the NESDA respondent had to meet diagnostic criteria for a depression or anxiety disorder either in the year before baseline or during the follow‐up, participated in at least two of the four previous waves and the current regular interview, and had the same biological parents as their sibling(s). The NESDA study, including the EMAA component, was approved by the VUmc ethical committee (reference number 2003/183) and all respondents gave informed consent for both the regular interview and the EMAA component.

**Figure 1:**
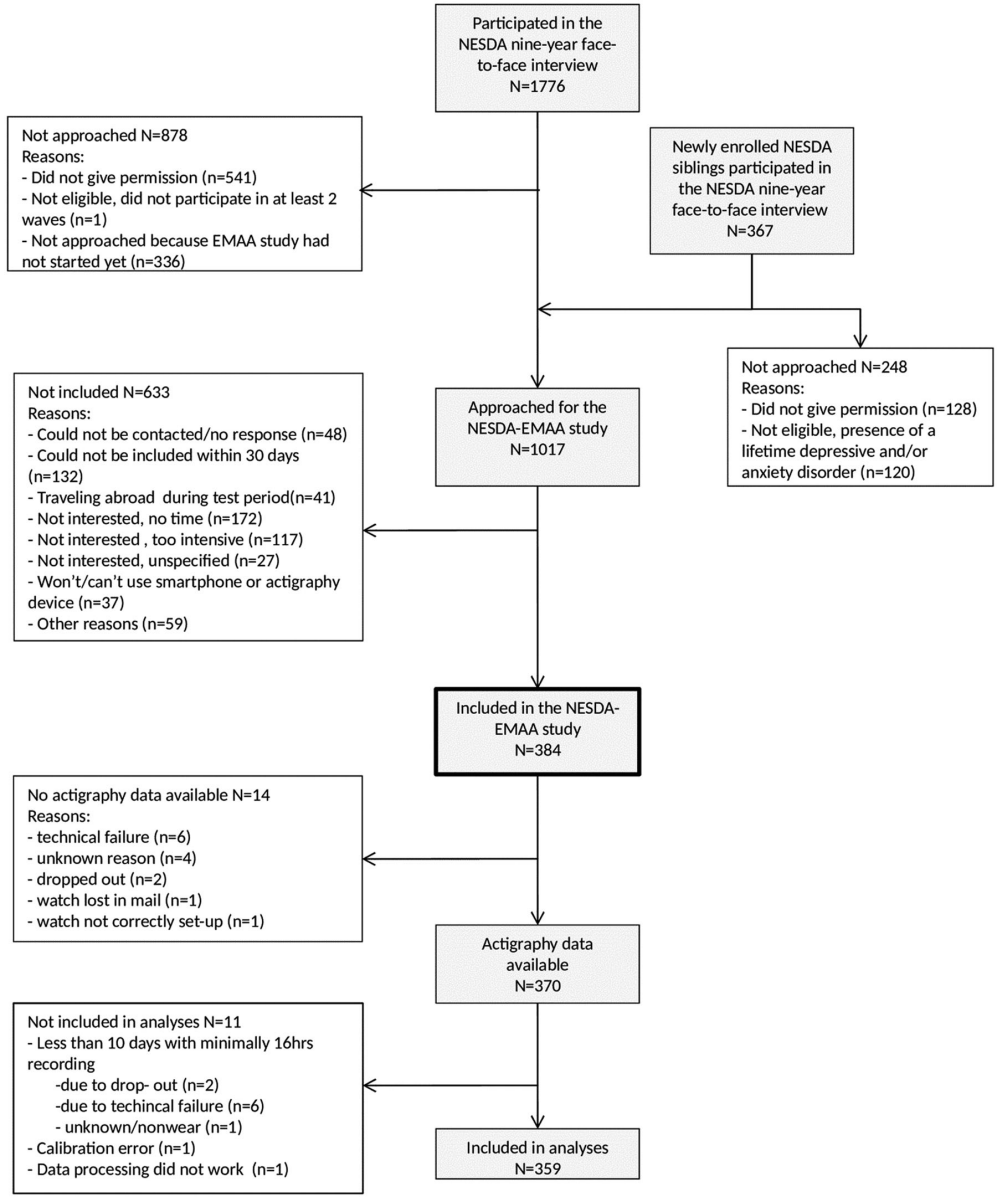
Flow‐chart NESDA‐EMAA study

A flowchart of the NESDA‐EMAA is provided in Figure 1. After the face‐to‐face 9‐year interview, participants of NESDA who were eligible and willing to participate in the NESDA‐EMAA sub‐study were invited to one of our research facilities within 1 month. For this study we invited NESDA participants who: participated in at least two of the previous NESDA waves, consented to be approached for this sub‐study, participated in the regular interview ≤ 31 days prior to starting the EMA measurements, had good mastery of the Dutch language, were familiar with smartphone use and willing to wear a wrist‐worn actigraphy device. Siblings were invited if they did not have a current or past diagnosis of a depressive and/or anxiety disorder or another severe psychiatric disorder (such as psychotic or severe addiction disorder). All participants were fully informed and given time to ask questions before participation. They received an instruction on the EMA and GENEActiv, and were provided with a GENEActiv device (Activinsights Ltd., Kimbolton, UK) as well as a prepaid envelope or box to send the GENEActiv back by mail after the 2‐week period. Participants wore the wrist‐worn GENEActiv actigraphy device and took part in the EMA assessment on a smartphone for 2 weeks during which they filled out questions on current mood states. If they did not have a smartphone with internet access, they could borrow one from the study. Of the 384 participants included in the NESDA‐EMAA study, 14 had no available actigraphy data for several reasons, such as technical failure (see Figure 1), resulting in 370 (96.4%) participants with available data. According to previously published criteria (da Silva et al., 2014), participant's actigraphy data were included in the analyses if at least 1 week day and 1 weekend day of usable data was available, with at least 16 hr recorded per day and per night (see Supporting Information “Raw data processing” for further details about the actigraphy data pipeline). The final sample was composed of 359 (93.5%) participants with 13.68 ± 1.26 valid days, of which 90% completed the protocol for 14 days.

### 2.2 Diagnosis of depression and/or anxiety disorders

As in the previous waves, at the 9‐year follow‐up, DSM‐IV–based diagnoses of depressive disorders (dysthymia and major depressive disorder) and anxiety (social anxiety disorder, panic disorder with and without agoraphobia, agoraphobia, and generalized anxiety disorder) were established with the composite international diagnostic interview (CIDI, version 2.1; Wittchen, 1994). The interviews were conducted by specially trained clinical research staff. For this study, participants were divided into the following three groups: (a) A group with no lifetime depressive and/or anxiety disorders, (b) a group with remitted depressive and/or anxiety disorders (having a lifetime, but not current [6‐month] diagnosis), and (c) a group with current depressive or anxiety disorder in the past 6 months.

### 2.3 Clinical characteristics

Studied clinical characteristics were the severity of depressive symptoms and that of anxiety symptoms, number of comorbid psychiatric disorders, duration of depressive or anxiety disorders, age of onset, and medication use (i.e., antidepressant and benzodiazepines use). Severity of depressive symptoms and that of anxiety symptoms were estimated using the 30‐item inventory of depressive symptomatology (IDS) self‐report (Rush, Gullion, Basco, Jarrett, & Trivedi, 1996) and the Beck anxiety inventory (BAI; Beck, Epstein, Brown, & Steer, 1988), respectively. Number of psychiatric disorders was determined as a count of current depressive and anxiety diagnoses at the 9‐year follow‐up. Duration of depressive or anxiety disorder was calculated as a count of the number of waves at which the patient reported a depression and/or anxiety diagnosis during the in‐between follow‐up periods (ranging from one to five waves). Age of onset was derived from the CIDI. Antidepressant use and benzodiazepine use was based on drug container inspection, and medications were coded according to the World Health Organization Anatomical Therapeutic Chemical (ATC) classification. Antidepressant and benzodiazepine use was considered present if participants reported using it more than 50% of the time. Antidepressants included were selective serotonin reuptake inhibitors (SSRIs, ATC code N06AB), tricyclic antidepressant (TCA, ATC code N06AA) and other antidepressants (ATC codes N06AF, N06AG, N06AX); benzodiazepines included ATC codes N03AE, N05BA, N05CD, and N05CF.

### 2.4 Actigraphy estimates of sleep, circadian rhythm, and physical activity

Participants wore the GENEActiv watch on their nondominant wrist for 2 weeks. They were instructed to wear the watch day and night and only taking it off when going to the sauna or when playing a contact sport in which wearing a wristband is unsafe. They were also instructed to press the button on the device when going to sleep and when getting up. In this study, the accelerometer was set to sample at 30 Hz and raw actigraphy data were analyzed using an open source R package, GGIR (version 1.5–18, see Supporting Information for further details on the raw actigraphy data cleaning processing pipeline). Sleep was assessed as total sleep duration per night (in hh:mm) and sleep efficiency per night (%). CR was assessed by mid sleep on free days (MSF; clock time) and the relative amplitude (RA) between day‐time and night‐time activity per day. Physical activity was assessed as gross motor activity per day (milli‐gravity [mg], 1*g* = 9.81 m/s^2^) and minutes in moderate‐to‐vigorous PA per day (objective minutes in moderate‐to‐vigorous PA per day were defined as the sum of 1‐min epochs in which gross motor activity was larger than 125 mg, which has recently been used by others (Kim et al., 2017)). Average weekly estimates were derived for each participant. Actigraphy variables were chosen based on several reasons. First, the selected actigraphy variables are among the ones reported frequently in adult studies and have been previously linked to psychopathology (Burton et al., 2013; Hori et al., 2016; Luik et al., 2015; Lyall et al., 2018). Second, these measures cover the concepts often collected with self‐reported questionnaires allowing for a comparison on their association with depressive and anxiety disorders.

### 2.5 Self‐reported sleep, CR, and PA

Self‐reported sleep was evaluated using the following questionnaires: Insomnia was assessed with the women's health initiative insomnia rating scale (IRS; Levine et al., 2003), which consists of five questions on difficulties in falling asleep, disruption and quality of sleep in the past month with a total score ranging from 0 (no insomnia) to 20 (severe insomnia). Sleep duration was also assessed (i.e., ≤6 hr, 7–9 hr, and ≥10 hr).

Self‐reported chronotype was assessed with the Munich chronotype questionnaire (MCTQ; Roenneberg, Wirz‐Justice, & Merrow, 2003). The MCTQ contains 29 questions about time of waking up and falling asleep on workdays and on free days, at this moment. Chronotype was defined as the midpoint in time between falling asleep and waking up on free days (MSF), since it is most likely to be accurate when one's natural CR can be observed, without the interference of work schedules and alarm clocks.

Self‐reported PA was assessed with the international PA questionnaire (IPAQ; Ainsworth et al., 2000). This seven‐item questionnaire provides information on the respondent's time spent on walking and on vigorous and moderate PA during the last 7 days. Minutes spent in moderate‐to‐vigorous PA and general PA, expressed as metabolic equivalent total (MET, 1 MET = 1 kcal·kg^−1^·hr^−1^) minutes per week were derived from the IPAQ. MET minutes per week were calculated as level × minutes of activity × events per week.

The reliability and validity of the used self‐reported questionnaires have been shown before in large‐cohort studies (IRS, Levine et al., 2003; MCTQ, Zavada, Gordijn, Beersma, Daan, & Roenneberg, 2005; and IPAQ, Craig et al., 2003).

### 2.6 Covariates

The covariates were age at the 9‐year follow‐up, sex and education level expressed in years. These covariates were selected as they have an established theoretical association with psychopathology and with sleep, CR, and PA levels, and have been regularly used in similar studies (Droomers, Schrijvers, & Mackenbach, 2001; Stamatakis, Kaplan, & Roberts, 2007).

### 2.7 Statistical analyses

Distributions of all variables were checked on normality with QQ plots. For descriptive statistics, participant demographics, clinical characteristics, actigraphy, and self‐reported estimates of sleep, CR, and PA were compared between the three groups (i.e., no, remitted, and current depressive and/or anxiety disorders). For normally distributed continuous data, analysis of variance (ANOVA) tests were used and for data that with a non‐normal distribution Kruskall‐Wallis tests were used. *χ* ^2^ tests were used to test differences in frequencies in the three groups. We calculated correlations between actigraphy and self‐reported estimates of sleep, CR, and PA. Correlations were tested with Pearson's correlation or with polyserial correlation tests when the variables were continuous or nominal‐continuous, respectively. This was done for the entire sample as well as stratified by the main grouping variable.

The association between actigraphy estimates and clinical characteristics (i.e., severity of depressive symptoms and severity of anxiety symptoms, number of psychiatric diagnoses, duration of psychiatric diagnoses, age of onset, and antidepressant use) was tested in separate models using linear regression model corrected for covariates and with each actigraphy estimate as outcome. Analyses to study the association with number of psychiatric diagnoses, duration of psychiatric diagnoses, age of onset, and antidepressant use were performed only including participants with current depressive and/or anxiety disorders. Non‐normally distributed outcomes were transformed with log‐transformation or with Box‐Cox transformation.

All analyses were performed with the statistical software R (version 1.0.143), a *p* < .05 was considered statistically significant. Posthoc tests were performed to allow multiple comparison for actigraphy estimates with Dunn's test when the differences between the three groups, that is, no, remitted, and current depressive and/or anxiety disorders were significant. To get a more comprehensive picture, we also performed posthoc analyses to check whether day‐to‐day variability in actigraphy estimates (calculated as the SD across 14 days) was associated with depressive and/or anxiety disorders.

The potential clustering of NESDA participants with their siblings was ignored, as of the 27 siblings included in the analyses, only seven were linked to NESDA participants in this sample. Analyses were rerun without the siblings that were linked to NESDA participants, but this did not alter results (data not shown).

## 3 RESULTS

The sample demographics and clinical characteristics are described in Table 1. Of the total sample (*n* = 359), 93 had current and 176 had remitted depressive and/or anxiety disorders, 90 had no current depressive and/or anxiety disorders. The current depressive/anxiety disorder group was heterogeneous: In that, 38.3% had anxiety disorders only, 33.0% had depressive and anxiety disorders, and 28.7% had depressive disorders only. As expected, persons with current depressive and/or anxiety disorders scored significantly higher on depressive and anxiety symptoms (both *p* < .001) and more frequently used antidepressant than both other groups, but no significant differences were found for benzodiazepine use.

**Table 1:** Demographic, psychiatric, psychological characteristics, and medication use in our NESDA sample (*n* = 359)

	Current depressive and/or anxiety disorders	Remitted depressive and/or anxiety disorders	No depressive and/or anxiety disorders	
*n*	93	176	90	*p*
Demographics				
Age, mean (*SD*)^a^	50.1 (11.1)	48.2 (13.4)	51.3 (12.5)	.13
Female, n (%)^b^	58 (62.4)	120 (68.2)	50 (55.6)	.12
Education level (years), mean (*SD*)	12.5 (3.4)	12.7 (2.8)	13.9 (2.9)	**<.001**
Psychopathology				
Only depressive disorders, n (%)	26 (28.7)	46 (26.1)	‐	
Only anxiety disorders, n (%)	36 (38.3)	24 (13.6)	‐	
Depressive & anxiety disorders, n (%)	31 (33.0)	106 (60.2)	‐	
Number of psychiatric disorders, median (IQR)	1 (1)	‐	‐	
Duration of psychiatric disorder/s (number of waves), median (IQR)	5 (1)	2 (2)	‐	
Age of depressive disorder or anxiety onset, mean (*SD*)	17.2 (12.2)	21.7 (12.9)	‐	
Psychological scales				
Inventory of depressive symptomatology (IDS), mean (*SD*)^a^	24.9 (13.2)	13.1 (8.9)	5.4 (3.8)	**<.001**
Beck anxiety inventory (BAI), mean (*SD*)^a^	13.6 (10.5)	6.1 (5.7)	1.6 (1.9)	**<.001**
Medication use				
Antidepressant users, n (%)^b^	35 (37.2)	34 (19.3)	2 (2.2)	**<.001**
Benzodiazepines users, n (%)^b^	5 (5.3)	8 (4.5)	0 (0.0)	.10

Abbreviations: IQR, interquartile range; *SD*, standard deviation

Bold value indicate *p* < .05.

^a^ Analysis of variance (ANOVA)

^b^ *χ* ^2^ test

When using actigraphy, persons with current depressive and/or anxiety disorders were significantly less active and had significantly lower RA between day‐time and night‐time activity compared to controls (*p* < .05, Table 2). We did not find significant differences for objective sleep duration and sleep efficiency. Self‐reported sleep, but not self‐reported PA or CR measures, differed significantly across groups (*p* < .001, Table 2). Low to moderate correlations were observed between self‐reported and objective estimates of sleep, CR, and PA (Table 3); objective and self‐reported sleep estimates were not significantly correlated, low but statistically significant, correlations were found between objective and self‐reported PA estimates, and moderate and significant correlations between objective and self‐reported CR estimates were observed. When stratifying by diagnostic status (i.e., no, remitted, and current depressive and/or anxiety disorders), we observed that correlations for mid sleep on free days were higher in the control group compared to patients with remitted and current depressive and/or anxiety disorders (Supporting Information, Table S1). On the other hand, correlations for PA estimates were no longer significant in people without depressive and/or anxiety disorders compared to the other groups (Supporting Information, Table S1). Our posthoc analyses on day‐to‐day variability in actigraphy estimates showed that patients with current depressive and/or anxiety disorders had significantly lower day‐to‐day variability in moderate‐to‐vigorous PA compared to controls (Supporting Information, Table S2).

**Table 2:** Actigraphy (white) and self‐reported (gray) estimates of physical activity, sleep, and circadian rhythm (*n* = 359)

	Current depressive and/or anxiety disorders	Remitted depressive and/or anxiety disorders	No depressive and/or anxiety disorders	*p*
Sleep				
Sleep duration (clock time), mean (*SD*)^b^	6:59 (1:08)	7:02 (0:59)	6:53 (0:58)	.478
Sleep duration^c^				.333
<7 hr, %	33.3	27.8	25.6	
7 ≤ hr ≤ 9, %	64.5	71.6	74.4	
>9 hr, %	2.2	0.6	0.0	
Sleep efficiency (%), median (IQR)^a^	87.0 (8.0)	88.0 (7.0)	88.0 (6.0)	.170
Self‐reported sleep duration^c^				**<.001** ^d^, ^e^
≤6 hr, %	40.9	19.9	13.3	
7 ≤ hr ≤ 9, %	53.8	76.7	86.7	
≥10 hr, %	5.4	3.4	0.0	
Insomnia rating scale, mean (*SD*)^b^	8.95 (4.74)	6.87 (4.46)	5.4 (3.52)	**<.001** ^d^, ^e^
Circadian rhythm				
Relative amplitude between day‐time and night‐time activity level, median (IQR)^a^	0.81 (0.08)	0.83 (0.06)	0.83 (0.06)	**.028** ^d^
Mid sleep on free days (clock time), median (IQR)^a^	04:10 (01:31)	04:11 (01:17)	04:14 (01:07)	.883
Self‐reported Mid Sleep on Free Days (clock time), mean (*SD*)^b^	04:03 (01:10)	03:55 (01:06)	04:00 (00:52)	.628
Physical activity				
Gross motor activity (milli‐gravity), median (IQR)^a^	23.80 (9.88)	26.90 (9.47)	27.40 (10.30)	**.021** ^d^
Moderate‐to‐vigorous physical activity (min/day), median (IQR)^a^	35.14 (35.43)	45.21 (44.32)	47.39 (41.91)	**.029** ^d^
Self‐reported moderate‐to‐vigorous physical activity (min/day), median (IQR)^a^	90 (139)	115 (120)	120 (120)	.061
Total MET‐min/week/1,000, median (IQR)^a^	2.19 (3.39)	2.85 (4.28)	2.60 (2.99)	.067

Abbreviations: IQR, interquartile range; MET, metabolic equivalent total; *SD*, standard deviation

Bold value indicate *p* < .05.

^a^ Kruskal‐Wallis test.

^b^ Analysis of variance (ANOVA).

^c^ *χ* ^2^ test.

^d^ Dunn's test, current depressive and/or anxiety disorders versus no depressive and/or anxiety disorders, *p* < .05.

^e^ Dunn's test, current depressive and/or anxiety disorders versus remitted depressive and/or anxiety disorders, *p *< .05.

**Table 3:** Correlations between self‐reported and actigraphy estimates of sleep, CR, and PA (*n* = 359)

	Self‐reported estimates				
	Sleep (IRS)		Circadian rhythm (MCTQ)	Physical activity (IPAQ)	
Actigraphy estimates	IRS^a^	Sleep duration^b^	Mid Sleep on Free Days^a^	MET^a^	MVPA^a^
Sleep					
Sleep duration	0.076	0.019	−0.017	−0.008	0.005
Sleep efficiency	−0.082	−0.043	−0.087	0.031	0.009
Circadian rhythm					
Relative amplitude between day‐time and night‐time activity level	−0.179***	0.231***	−0.140**	0.235***	0.146**
Mid sleep on free days	−0.078	0.075	0.582***	−0.025	−0.017
Physical activity					
Gross motor activity	−0.113*	0.105	−0.028	0.286***	0.203***
MVPA	−0.089	0.134	0.009	0.258***	0.177***

Abbreviations: IPAQ, international physical activity questionnaire; IRS, women's health initiative insomnia rating scale; MCTQ, Munich chronotype questionnaire; MET, metabolic equivalent total; MVPA, moderate‐to‐vigorous physical activity

^a^ Pearson's correlation.

^b^ Polyserial correlation.

* *p* < .05.

** *p* < .01.

*** *p* < .001.

Having higher levels of depressive and anxiety symptoms was significantly associated with longer sleep duration, lower RA between day‐time and night‐time activity level, reduced gross motor activity, and moderate‐to‐vigorous PA (Table 4). Looking at the smaller sized group of 93 persons with current depressive and/or anxiety disorders, number of psychiatric diagnoses was the only clinical characteristic significantly associated with longer sleep duration, lower RA between day‐time and night‐time activity level, lower gross motor activity, and moderate‐to‐vigorous PA Table 4). Antidepressant use was significantly associated with delayed mid sleep on free days (Table 4).

**Table 4:** Associations* between actigraphy‐derived sleep, CR, and PA and clinical characteristics

	Sleep duration			Sleep efficiency			Relative amplitude			Mid sleep on free days			Gross motor activity			MVPA		
	Β	SE	*p*	β	SE	*p*	β	SE	*p*	β	SE	*p*	β	SE	*p*	β	SE	*p*
Psychological scales (*n* = 359)																		
IDS	0.162	0.053	**.002**	−0.084	0.053	.115	−0.205	0.052	**<.001**	0.132	0.054	**.016**	−0.239	0.051	**<.001**	−0.185	0.051	**<.001**
BAI	0.141	0.053	**.008**	−0.033	0.054	.544	−0.154	0.052	**.003**	0.069	0.055	.206	−0.212	0.051	**<.001**	−0.169	0.051	**.001**
Clinical characteristics among current cases (*n* = 93)																		
Number of psychiatric diagnoses	0.309	0.100	**.003**	−0.038	0.104	.714	−0.310	0.102	**.003**	0.131	0.108	.227	−0.383	0.099	**<.001**	−0.305	0.100	**.003**
Duration of psychiatric diagnoses (waves)	0.129	0.110	.241	0.060	0.109	.584	−0.028	0.112	.803	0.113	0.113	.323	−0.106	0.112	.346	−0.037	0.111	.738
Age of onset	−0.172	0.112	.127	−0.029	0.112	.798	0.074	0.115	.519	0.07	0.117	.548	0.102	0.115	.375	0.004	0.114	.970
Antidepressant use	0.169	0.213	.430	−0.198	0.210	.348	−0.271	0.215	.211	0.545	0.212	**.012**	−0.344	0.214	.111	−0.343	0.211	.108

Abbreviations: BAI, Beck anxiety inventory score; IDS, inventory of depressive symptomatology score; MVPA, moderate‐to‐vigorous physical activity; RA, relative amplitude between day‐time and night‐time activity level

Bold value indicate *p* = .012.

* Linear regression model adjusted for age, sex, and education. Log transformation was applied to gross motor activity and mid sleep on free days. Box‐Cox transformation was applied to MVPA, sleep efficiency, and RA.

## 4 DISCUSSION

In this study, we found reduced PA level and daily rhythm disturbances among those with depressive and anxiety disorders compared to controls using objective measures, but not using self‐reported information. By contrast, self‐reported but not objective sleep measures differed between persons with depressive and anxiety disorders versus controls. Actigraphy estimates gathered in an ecologically valid manner during continuous registration over 2‐week time revealed objective differences in PA and CR measures between diagnostic groups that were not captured with self‐reported questionnaires. Moreover, self‐reported sleep appeared to reflect the sleep misperception commonly presented in persons with depressive and anxiety symptoms (Dittoni et al., 2013). This supports the use of actigraphy in the assessment of PA, sleep, and CR disturbances in epidemiological studies and clinical practice enhancing subjective data from the traditional self‐reported questionnaires. The high participation response to the study confirms the feasibility and acceptability of monitoring patients with depressive and anxiety disorders with actigraphy, which may translate to the clinical setting as well.

We found lower levels of objective PA (and lower day‐to‐day variability in objective PA) in persons with a current diagnosis, versus those with remitted or no diagnosis. Posthoc analysis (data not shown) confirmed that associations for pure anxiety and pure depression cases were similar. Patients with current depression and anxiety spent on average 35 min per day in moderate‐to‐vigorous PA and a similar effect size has been found in the meta‐analysis on depression by Schuch et al. (2017). In terms of the association between CR disturbances with psychopathology, we found that depressive and anxiety disorders are associated with lower RA between day‐time and night‐time activity levels. Similarly, previous studies have shown that CR appears to be dampened in patients with depression (Hori et al., 2016) and mood disorders (Lyall et al., 2018; Shou et al., 2017), suggesting that the lower level of daily activity is a core feature of mood disorders (Burton et al., 2013). Importantly, less research has been conducted for anxiety, however, our results showed similar effects to those in depression. Reduced daily activity level and CR amplitude may be indicative of psychomotor retardation, withdrawal from normal activities of daily living (Burton et al., 2013) and circadian impairments (Lyall et al., 2018). As improvement in depression with antidepressant medications have been linked with greater day‐time activity levels (Todder, Caliskan, & Baune, 2009) and higher RA between day‐time and night‐time activity (Todder et al., 2009) when using actigraphy, the continuous measurement of daily activity and circadian rhythmicity with wrist‐worn actigraphy may help to monitor treatment effects (Martin & Hakim, 2011).

However, first some conditions should be met before actigraphy could play a role in monitoring of treatment response. Although it has been suggested that actigraphy could be used to follow the progression of depression treatment, it is still unclear how sensitive and specific actigraphy is for the characterization of this change in psychological state (Martin & Hakim, 2011). Which actigraphy variables are best suited for monitoring purposes and definitions of clinically relevant improvement on these variables needs to be examined. Interpretation is also not always immediate (e.g., RA between day‐time and night time activity) and clinicians may need additional training for the use of devices in the clinical practice. Wearing research devices such as a GENEActiv device, may be experienced as stigmatizing as a patient may be asked for its use in public (Scott et al., 2019; Simblett et al., 2019). Popular commercial activity trackers are aesthetically pleasing and therefore maybe less stigmatizing, but at a cost of lower quality data—whether these devices are suitable for monitoring treatment effects is unknown (Scott et al., 2019). Currently, the majority of devices used in research settings need to be connected to a computer to download data and to extract variables with the use of specific software, resulting in a time‐consuming procedure. In the future, devices with bluetooth‐compatible connectivity and consumer devices are likely to become more common, allowing the real‐time synchronization of data on the patient's smartphone app and on a data visualization dashboard. Data may become readily available and interpretable for the clinician, while for the patient, real‐time feedback on activity and sleep may help to adopt more healthy habits (Matcham et al., 2019). While actigraphy appeared to enhance information obtained from self‐reported questionnaires, self‐reported assessment can also be improved by using a daily electronic diary that is less costly and time consuming than actigraphy and it may be more personalized to individuals (Martin & Hakim, 2011). Such electronic diaries may also incorporate other questionnaires on sleep and sleep quality such as the Pittsburgh sleep quality index or the insomnia severity index.

Actigraphy indices of sleep were not significantly different across diagnostic groups. Moreover, while sleep duration measured with actigraphy was not associated with depression and anxiety, self‐reported sleep duration was consistently associated with psychopathology in the current study and elsewhere (Luik et al., 2015). Self‐reported and objectively assessed sleep duration also appeared to be poorly correlated, and this possibly indicates that perceived sleep reflects different aspects of sleep than the objective estimate. This might be due to sleep misperception, which is more common in patients with severe depressive and anxiety symptoms (Dittoni et al., 2013), and is influenced by their negative perception. It has been argued (Harvey & Tang, 2013) that this tendency to misperceive sleep does not preclude the presence of a real sleep deficit. Perhaps, the increased worry associated with insomnia makes it more difficult for patients to move into the deeper stages of sleep and the lighter stages of sleep may be more likely to be perceived as wake (Harvey & Tang, 2013). It should be also mentioned that the tendency to misperceive sleep may be considered as a “prodromic or transitional state” in the development of insomnia that is characterized by a serious objective sleep deficit (Harvey & Tang, 2013). Thus, actigraphy may be used to determine whether self‐reported sleep problems are not consistent with objective sleep, and the patient would potentially benefit from psychologic treatments to reverse misperception, such as cognitive behavioral therapy (Martin & Hakim, 2011).

We also found evidence for dose‐response associations as those with more severe depressive and anxiety symptoms were significantly less active and showed longer sleep duration and lower RA between day and night activity level. Furthermore, in the group with current depression and/or anxiety, we found that psychiatric comorbidity was associated with physical inactivity, longer sleep duration, and lower RA between day and night activity level. As PA and CR are modifiable factors, exercise, behavioral activation, and chronotherapy may be offered as adjunctive treatments to usual care in these groups of patients (Carek, Laibstain, & Carek, 2011; Chum et al., 2017; Morgenthaler et al., 2007). The dose‐response relationships provide further evidence for the ecological validity of actigraphy. Although, recent clinical trials have employed actigraphy to monitor activation therapy (Averill et al., 2019) and sleep deprivation (Arnedt et al., 2017) as adjunctive treatments to usual care, or as a tool to complement behavioral activation (Chum et al., 2017), more research is needed to clarify whether actigraphy may be used to monitor treatment outcomes of such interventions.

Sleep duration was found to be longer in persons with a greater number of depressive and anxiety symptoms. This can be explained by the fact that hypersomnia is one of the symptoms of depression and it usually characterizes patients with atypical features. Another explanation may be antidepressant use. More severe cases are more likely to use these medications, and, previous research has suggested that long sleep duration can be a result of antidepressant use (Robillard et al., 2015), such as sedating tricyclic antidepressants (Wichniak & Jernajczyk, 2017). While benzodiazepine use could also be an explanation, as it is also prescribed for sleep problems, it is a less likely explanation here, as only five cases used benzodiazepines, and analyses excluding these cases did not alter findings (data not shown). Another possible explanation of our findings is that the used sleep detection algorithm may misclassify period of inactivity as sleep period, which may be possible since patients with depression and anxiety exhibit more sedentary behavior.

Shifted and delayed rhythm have been previously documented in patients with depression (Hori et al., 2016) and anxiety (Robillard et al., 2015) supporting the evidence for an association between late chronotypes and psychopathology. In our sample, we noticed later mid sleep on free days among current cases using antidepressant medication after covariate adjustments. As antidepressant use may result in more day‐time sleepiness and fatigue (Fava, 2003) and longer sleep duration (Robillard et al., 2015), this may possibly explain this finding.

Some limitations should be considered in the current study. Although actigraphy provides an estimate of sleep parameters such as sleep duration, sleep efficiency, it does not yield estimates of sleep stage, such as REM period that may be associated with depression. In addition, it was not possible to extrapolate actigraphy sleep estimates indicative of insomnia such as sleep‐onset latency (i.e., time to transit from full wakefulness to sleep) and wake after sleep onset (i.e., periods of wakefulness occurring after sleep onset). Studies have also shown that actigraphy lacks the precision of the gold standard polysomnography that provides greater insight into sleep problems in people with depression or anxiety (Van Hees et al., 2015). Because sleep is inferred from a lack of movement, there may be some misclassification of sleep among those who are awake but motionless. Therefore, the technique is biased toward overestimating total sleep duration, which may lead to incorrectly minimizing the severity of sleep disturbances. Likewise, assessment of CR with actigraphy may not reflect the underlying circadian biology and may be influenced by unmeasured demographics and other lifestyle factors (Luik, Zuurbier, Hofman, Van Someren, & Tiemeier, 2013). We also based our threshold for MVPA on that of other studies (Kim et al., 2017), but there is no consensus on these thresholds. We also used a less frequently reported measure of chronotype (sleep midpoint on free days). Chronotype is often represented as sleep midpoint on free days corrected for sleep‐debt accumulated during working days (MSFsc; Roenneberg et al., 2003). As we did not find associations with MSF and MSF and MSFsc were previously found to be highly correlated in the NESDA sample (r = 0.91; Antypa et al., 2016), we did not expect to find different results in our analysis and the conclusions would not change. Nevertheless, the overall chronotype (early, intermediate, and late chronotype) across a sample may influence any correlational associations leading to inconsistent associations with psychopathology (Dimitrov et al., 2018). It should also be noted that the self‐reported questionnaires and the actigraphy measurement did not capture the same time period; questionnaires were filled out before actigraphy. We also used a limited number of sociodemographic covariates in this study (i.e., age, sex, education). Future analyses may take other variables into account (e.g., BMI, employment status) to study what factors may explain the association of sleep, CR, and PA with psychopathology. Correction for multiple testing was also not performed and may have led to finding statistical associations for chance. Strengths of the study are the inclusion of both depressive and anxiety disorder, and the ability to look in‐depth at the role of clinical characteristics.

While the measures presented in this paper provide some preliminary insight into the objectively measured PA, sleep, and CRs and the differences therein between diagnostic groups, these measures are summaries of a much richer underlying data set. Using studies from our collaborative Motor Activity Research Consortium for Health (m‐MARCH) initiative (http://www.mmarch.org, Scott et al., 2017), we plan to employ more sophisticated analytical tools have been developed that can improve our understanding of these features, such as functional principal component analyses (Gershon, Ram, Johnson, Harvey, & Zeitzer, 2016), and functional scalar regression (Goldsmith, Zipunnikov, & Schrack, 2015). These approaches will enhance the power and gain greater insight into these patterns and their relationship to depression and anxiety disorders.

To conclude, we found that persons with current depression and/or anxiety exhibit reduced PA and more CR disturbances than controls using actigraphy. As the correlations of actigraphy estimates with self‐report measures were generally low, actigraphy monitoring was shown to provide an easy and noninvasive approach to capture objective information regarding both night time sleep and day‐time activity, and, sleep and CR. In addition, persons with greater severity and, among current cases, with more psychiatric comorbidity showed lower PA and more CR disturbances. Therefore, adjunctive behavioral and chronotherapy interventions in depression and anxiety may especially focus on these individuals. While this study confirms the feasibility and acceptability of monitoring patients, more research is needed to establish whether actigraphy could, possibly, in the future, play a role in monitoring treatment response to such interventions.

## DISCLOSURE

Sonia Difrancesco reports grants from Innovative Medicines Initiative 2 Joint, during the conduct of the study; Femke Lamers reports grants from European Union Seventh Framework Programme (FP7/2007–2013), during the conduct of the study; Brenda W. J. H. Penninx reports grants from Dutch government, Ministry of Health, NOW, during the conduct of the study; and Brenda W. J. H. Penninx had received (unrelated) funding for her research from Boehringer Ingelheim and Janssen pharmaceuticals. Other coauthors have nothing to declare.

## DATA AVAILABILITY

According to European law (GDPR) data containing potentially identifying or sensitive patient information are restricted; our data involving clinical participants are not freely available in a public repository. However, data are available upon request via the NESDA Data Access Committee (nesda@ggzingeest.nl).

## Supporting information

Supporting information
