# Supplementary material for: Sleep, circadian rhythm, and physical activity patterns in depressive and anxiety disorders: A 2‐week ambulatory assessment study
Source: Depress Anxiety. 2019 Jul 26;36(10):975–86. doi: 10.1002/da.22949 (PMC6790673; doi:10.1002/da.22949)
Supplement: Supplementary file 1 — Supporting information [file DA-36-975-s001.docx]

**Supplemental material**

**Raw data processing**

We used the open source R package, GGIR (version 1.5-18), for cleaning the raw actigraphy data. According to previously published methods (van Hees et al., 2014), the detailed processing pipeline included the following steps: verification of sensor calibration error using local gravity as a reference, detection of sustained abnormally high values, non-wear detection and, extraction of objective physical activity, sleep and circadian rhythm measures. Of those 370 participants with available data, one participant (0.3%) had calibration error higher than 0.02 g (1g = 9.81m/s^2^), one participant (0.3%) had sustained abnormally high values, and therefore excluded. The processing pipeline implemented by GGIR failed to process data of one participant for unknown reasons.

As participants were found to inconsequently comply to the protocol to press the button when going to sleep/getting up (2 participants, 0.5%), a new method was used to calculate sleep estimates (i.e., total sleep duration per night and sleep efficiency per night) without the use of a sleep diary (van Hees et al., 2018). Inactivity periods were defined as consecutives 5-second epochs in which the arm angle relative to the horizontal plane did not change of more than 5° over at least 5 minutes (Van Hees et al., 2015). With a heuristic algorithm, a Sleep Period Time-window was identified as the time window starting at sleep onset and ending when waking up after the last sleep episode of the night (van Hees et al., 2018). Inactivity periods overlapping the Sleep Period Time-window were labelled as sleep periods. Finally, total sleep duration [clock time] was calculated as the sum of estimated sleep periods and sleep efficiency [%] was the total sleep duration divided by the time difference between sleep onset and wake-up time (i.e., time in bed).

Circadian rhythm was calculated by Mid Sleep on Free Days and the relative amplitude between daytime and night-time activity. Assuming that weekend days are most likely to be free days of the week, Mid Sleep on Free Days was calculated as the middle time point between sleep onset and wake-up time during weekend days. Relative amplitude between daytime and night-time activity was calculated according to previously published methods (Van Someren et al., 1999). Average weekly estimates were derived as [(average value for weekdays × 5 + average value for weekend days × 2) / 7], while the average was calculated for Mid Sleep on Free Days.

Physical activity was assessed as gross motor activity per day and minutes in moderate-to-vigorous physical activity per day. Objective gross motor activity was estimated by calculating the Euclidian Norm Minus One (ENMO: $\sqrt{x^{2}+ y^{2}+ z^{2}}-1g$, 1g = 9.81m/s^2^) with any negative values rounded up to zero and by averaging such measure over 5-second epoch (van Hees et al., 2013). Because there is no consensus on thresholds to identify moderate-to-vigorous physical activity, objective minutes in moderate-to-vigorous physical activity per day were defined as the sum of 1-min epochs in which ENMO was larger than 125mg, which has recently been used by others (Kim et al., 2017).

Table S1: Correlations between self-reported and actigraphy estimates of sleep, circadian rhythm and physical activity stratified by diagnostic status (n=359)

|  | **No depressive and/or anxiety disorders (n =90)** | | | | | **Remitted depressive and/or anxiety disorders (n=176)** | | | | | **Current depressive and/or anxiety disorders (n=93)** | | | | |
| --- | --- | --- | --- | --- | --- | --- | --- | --- | --- | --- | --- | --- | --- | --- | --- |
|  | **Self-reported estimates** | | | | | **Self-reported estimates** | | | | | **Self-reported estimates** | | | | |
|  | **Sleep (IRS)** | | **Circadian rhythm (MCTQ)** | **Physical activity (IPAQ)** | | **Sleep (IRS)** | | **Circadian rhythm (MCTQ)** | **Physical activity (IPAQ)** | | **Sleep (IRS)** | | **Circadian rhythm (MCTQ)** | **Physical activity (IPAQ)** | |
| **Actigraphy estimates** | IRS ^a^ | Sleep duration ^b^ | Mid Sleep on Free Days ^a^ | MET ^a^ | MVPA ^a^ | IRS ^a^ | Sleep duration ^b^ | Mid Sleep on Free Days ^a^ | MET ^a^ | MVPA ^a^ | IRS ^a^ | Sleep duration ^b^ | Mid Sleep on Free Days ^a^ | MET ^a^ | MVPA ^a^ |
| **Sleep** |  |  |  |  |  |  |  |  |  |  |  |  |  |  |  |
| Sleep duration | 0.149 | 0.198 | 0.028 | 0.001 | 0.013 | 0.076 | 0.022 | 0.039 | -0.035 | 0.033 | 0.012 | -0.038 | -0.131 | 0.030 | -0.062 |
| Sleep efficiency | -0.063 | -0.067 | -0.047 | 0.043 | 0.061 | -0.05 | -0.029 | -0.077 | 0.001 | -0.001 | -0.112 | -0.142 | -0.119 | 0.045 | -0.059 |
| **Circadian rhythm** |  |  |  |  |  |  |  |  |  |  |  |  |  |  |  |
| RA | -0.016 | 0.015 | -0.380*** | 0.205* | 0.156 | -0.247*** | 0.314*** | -0.110 | 0.261*** | 0.186* | -0.051 | 0.082 | -0.054 | 0.180 | 0.013 |
| Mid Sleep on Free Days | 0.087 | -0.063 | 0.638*** | 0.021 | -0.005 | -0.069 | 0.056 | 0.604*** | 0.019 | 0.025 | -0.220* | 0.174 | 0.508*** | -0.163 | -0.117 |
| **Physical activity** |  |  |  |  |  |  |  |  |  |  |  |  |  |  |  |
| Gross motor activity | -0.023 | 0.070 | -0.292** | 0.163 | 0.126 | -0.187* | 0.113 | 0.048 | 0.285*** | 0.204** | 0.081 | -0.005 | 0.024 | 0.379*** | 0.219* |
| MVPA | 0.029 | 0.108 | -0.278** | 0.126 | 0.092 | -0.145 | 0.093 | 0.082 | 0.259*** | 0.182* | 0.081 | 0.086 | 0.106 | 0.369*** | 0.190 |

Abbreviations: IPAQ, International Physical Activity Questionnaire – Short Form; IRS, Women’s Health Initiative Insomnia Rating Scale; MCTQ, Munich Chronotype Questionnaire; MET, metabolic equivalent total; MVPA, moderate-to-vigorous physical activity; RA, relative amplitude between daytime and night-time activity level.

Notes: *: p < 0.05; **: p < 0.01; ***: p < 0.001.

^a^ Pearson’s correlation

^b^ Polyserial correlation

**Table S2: day-to-day variability in sleep, circadian rhythm and physical activity in persons with current, remitted, no depressive and/or anxiety disorders (n = 359)**

|  | **Current depressive and/or anxiety disorder(s)** | **Remitted depressive and/or anxiety disorder(s)** | **No depressive and/or anxiety disorder(s)** | **p** |
| --- | --- | --- | --- | --- |
| **Sleep** |  |  |  |  |
| Sleep duration variability [clock time], mean(sd) ^a^ | 01:05 (00:28) | 01:10 (00:37) | 00:59 (00:25) | **0.045** ^b^ |
| Sleep efficiency variability [%], mean (sd) ^a^ | 0.05 (0.03) | 0.05 (0.03) | 0.05 (0.02) | 0.234 |
| **Circadian rhythm** |  |  |  |  |
| Variability in relative amplitude between day and night activity, mean (sd) ^a^ | 0.05 (0.04) | 0.05 (0.04) | 0.05 (0.03) | 0.661 |
| Mid Sleep on Free Daysvariability [clock time], mean (sd) ^a^ | 00:38 (00:34) | 00:41 (0:43) | 00:37 (00:31) | 0.297 |
| **Physical activity** |  |  |  |  |
| Gross motor activity variation [milli-gravity], mean (sd) ^a^ | 4.73 (2.45) | 5.1 (3.06) | 5.51 (3.51) | 0.197 |
| Moderate-to-vigorous physical activity variability [min], mean (sd) ^a^ | 18.02 (15.81) | 21.46 (18.28) | 25.51 (17.56) | **0.015** ^c^ |

^a^ Kruskal–Wallis test;

^b^ Dunn’s test, remitted depressive and/or anxiety disorders versus no depressive and/or anxiety disorders, p<0.05

^c^ Dunn’s test, current depressive and/or anxiety disorders versus no depressive and/or anxiety disorders, p<0.05

**References**

Kim, Y., White, T., Wijndaele, K., Sharp, S. J., Wareham, N. J., & Brage, S. (2017). Adiposity and grip strength as long-term predictors of objectively measured physical activity in 93 015 adults: the UK Biobank study. *International Journal of Obesity*, *41*, 1361–1368.

van Hees, V. T., Fang, Z., Langford, J., Assah, F., Mohammad, A., da Silva, I. C. M., … Brage, S. (2014). Autocalibration of accelerometer data for free-living physical activity assessment using local gravity and temperature : an evaluation on four continents. *J Appl Physiol*, (117), 738–744. https://doi.org/10.1152/japplphysiol.00421.2014

van Hees, V. T., Gorzelniak, L., Leon, E. C. D., Eder, M., Pias, M., Taherian, S., … Brage, S. (2013). Separating Movement and Gravity Components in an Acceleration Signal and Implications for the Assessment of Human Daily Physical Activity. *PLOS ONE*, *8*(4), 1–10. https://doi.org/10.1371/journal.pone.0061691

Van Hees, V. T., Sabia, S., Anderson, K. N., Denton, S. J., Oliver, J., Catt, M., … Singh-Manoux, A. (2015). A novel, open access method to assess sleep duration using a wrist-worn accelerometer. *PLoS ONE*, *10*(11), 1–13. https://doi.org/10.1371/journal.pone.0142533

van Hees, V. T., Sabia, S., Jones, S. E., Wood, A. R., Anderson, K. N., Kivimaki, M., … Weedon, M. N. (2018). Estimating sleep parameters using an accelerometer without sleep diary. *BioRxiv*.

Van Someren, E. J. W., Swaab, D. F., Colenda, C. C., Cohen, W., McCall, W. V., & Rosenquist, P. B. (1999). Bright Light Therapy: Improved Sensitivity to Its Effects on Rest-Activity Rhythms in Alzheimer Patients by Application of Nonparametric Methods. *Chronobiology International*, *16*(4), 505–518. https://doi.org/10.3109/07420529908998724
